# Update on Pediatric Hepatitis C Infection

**DOI:** 10.1007/s11894-024-00955-3

**Published:** 2025-02-28

**Authors:** Johanna Ferreira, Shari Sheflin-Findling

**Affiliations:** Division of Pediatric Gastroenterology, Liver Disease and Nutrition, Cohen Children’s Medical Center/Northwell, The Zucker School of Medicine at Hofstra, 1991 Marcus Avenue, Suite M100 , Lake Success, NY 11042 USA

**Keywords:** Hepatitis C infection, Hepatitis, Liver disease, Direct-acting antiviral agents, Pharmacotherapy

## Abstract

**Purposeof Review:**

Hepatitis C virus (HCV) infections continue to steadily increase in the United States and remain a major public health challenge. This review aims to provide a comprehensive overview of HCV infection in children, focusing on recent advancements in screening, diagnosis, and treatment.

**Recent Findings:**

Effective screening strategies, including universal screening of pregnant women and nucleic acid testing for all perinatally exposed infants at 2 to 6 months of age, have been implemented to identify infected individuals early. Direct-acting antiviral agents have revolutionized treatment, offering high cure rates for children of all ages.

**Summary:**

Despite significant progress, challenges remain in achieving HCV elimination. These include the need for improved access to testing and treatment, as well as ongoing efforts to develop a preventive vaccine. Continued research and implementation of effective strategies are essential to reduce the burden of HCV infection.

## Introduction

Hepatitis C virus (HCV) infections continue to steadily increase in the United States and remain a major public health challenge. The rate of acute HCV infection has tripled among reproductive-aged persons in the United States as of 2021, from 0.8 to 2.5 per 100,000 population among persons aged 20–29 years and from 0.6 to 3.5 among persons aged 30–39 years [1]. Globally, an estimated 50 million people have chronic HCV infection, with about one million new infections occurring per year as per the World Health Organization (WHO) [2]. The WHO estimated that in 2022, approximately 242,000 people died from hepatitis C, mostly from cirrhosis and hepatocellular carcinoma. HCV is transmitted through percutaneous exposure to infected blood; increases in infection rates have correlated with the opioid and injection drug use (IDU) epidemic [3–5]. Unfortunately, unrecognized cases do not get reported to public health authorities and current statistics may underrepresent the actual public health burden. Fortunately, an effective cure for hepatitis C is now available with the development of new antiviral medications, the direct-acting antiviral agents (DAA). With a cure at hand, the WHO has set a goal of eliminating HCV by 2030 [6]. The challenges to meet this goal include the implementation of effective screening strategies and closing the testing to treatment gap.

## Hepatitis C Virology, Epidemiology and Transmission

### Virology

HCV, a single-stranded RNA virus within the Flaviviridae family, exhibits significant genotypic diversity. At least seven distinct genotypes, further classified into subtypes, are known to infect humans. These genotypes display marked geographic variation and influence treatment outcomes. Globally, genotypes 1, 2, 3, and 4 account for the majority of HCV infections. Genotype 1, the most prevalent worldwide, is also the dominant strain in the United States and Europe, encompassing approximately half of all cases. South Asia exhibits the highest prevalence of genotype 3, while East Asia is a hotspot for genotypes 2 and 6. North Africa and the Middle East are characterized by a predominance of genotype 4 infections [7]. Notably, genotype 7, a novel HCV lineage, was first identified in the Democratic Republic of Congo in 2015 [8].

### Epidemiology and Transmission

Injection drug use (IDU) is the most reported risk factor among adults for acquiring HCV infection. Data reported by WHO from 1995–2020 for 105 countries estimated that unsafe IDU practices contribute to 43.6% (33.9% – 52.5%) of new HCV infections globally [2]. Ten countries are estimated to account for nearly 80% of all global HCV infections among people who inject drugs, with United States and China leading this list.

For young children, perinatal transmission is the primary risk factor. In 2018 WHO estimated 3.26 million children and adolescents living with chronic HCV infection. Other modes of transmission include sexual contact, health care procedures, needlestick injuries in health care settings, unregulated tattooing, and sharing personal items contaminated with infectious blood. With the recent opioid epidemic in the United States, there have been increasing cases of HCV in both adult and adolescent populations; as such chronic cases are now highest among reproductive-age persons from 20–39 years [1].

HCV infections during pregnancy have increased by 20% during 2016–2020 and up to tenfold during 2000–2019 [5, 9]. Seroprevalence of HCV during pregnancy is estimated to be about 1.2% [10]. Recent data from a meta-analysis and a systematic review demonstrated that the risk of perinatally acquired infection from an HCV antibody-reactive pregnant woman with detectable HCV RNA is 5.8%−7.2% in the absence of human immunodeficiency virus (HIV) infection and 10.8%−12.1% among those with poorly controlled HIV coinfection [11, 12]. The risk of HCV perinatal transmission among HIV coinfected pregnant woman is reduced by lowering the HIV viral load during pregnancy.

In 2021, a total of 199 perinatal HCV cases were reported to the Centers for Disease Control and Prevention (CDC) from 28 states, with approximately one half of cases reported by six states. This approximation is likely a substantial underestimate of the number of cases in the United States. Several factors have been linked to an increased risk of mother-to-child transmission (MTCT) of HCV, including prolonged membrane rupture (> 6 h) before delivery, use of internal fetal monitoring, and high maternal HCV viremia during pregnancy [13, 14].

However, it is important to note that a negative HCV viral load does not guarantee the absence of MTCT risk. Cesarian delivery is not recommended over vaginal delivery to prevent perinatal transmission. Moreover, breastfeeding does not increase the risk of HCV transmission and should not be contraindicated. The CDC recommends against breastfeeding if there are bleeding or cracked nipples [10].

### Natural History

Spontaneous clearance of HCV occurs more frequently in children who acquired the infection perinatally compared to adolescents or adults who acquired the virus by other routes. Of children infected via perinatal transmission, 20%−40% have resolution of the infection resulting in undetectable virus by 5 years of age [13, 15, 16]. The majority of these occur within the first 2 years of life. A more recent study has shown spontaneous clearance of these infections at an estimated 65.9% by 5 years of age [17]. In contrast, adolescents and adults progress to chronic infection at a rate of over 70% [18, 19]. Early viral clearance may be associated with a higher alanine aminotransferase (ALT) level in children under two years of age, compared to those who do not achieve clearance [20]. This observation warrants further investigation to determine the underlying mechanism. Spontaneous viral clearance in HCV is influenced by both host and viral factors. On the viral side, genotype 3 HCV and the presence of the interleukin 28B rs12979860 single-nucleotide polymorphism (SNP) have been associated with increased clearance rates. Host factors in both mothers and children, including human leukocyte antigen (HLA) class I/II alleles, killer-cell immunoglobulin-like receptor (KIR) genes, and KIR-ligand-binding polymorphisms, are also believed to play a role. Furthermore, significant differences in circulating natural killer (NK) cells (CD56 + CD3) and other lymphocyte phenotypes observed in children with chronic HCV infection compared to healthy controls highlight the potential importance of variations in the immune response for determining disease outcome in children [21].

HCV infection is characterized by an asymptomatic and slowly progressive course over several decades. This high prevalence of asymptomatic infection likely leads to underestimation of the true public health burden imposed by HCV, highlighting the critical need for effective screening strategies. Symptomatic presentations, when they occur, are characterized by non-specific features such as fatigue, fever, and myalgia in acute cases, and fatigue, abdominal pain and hepatomegaly in chronic cases. HCV‐induced fulminant hepatic failure is infrequent [22]. Children with chronic HCV can present with elevations in serum aminotransferase levels, suggesting a gradual progression of liver disease over several decades. As further support of this gradual progression, the degree of inflammation on liver biopsy appears to correlate with duration of disease [23]. Hepatomegaly occurs in about 10% of children and is associated with elevated ALT levels [20, 24].

The lifelong risk of progression to cirrhosis and advanced liver disease for HCV acquired perinatally is not well established. Rates as high as 30% were reported in one study with a median duration of 33 years [25]. Overall progression is uncommon before adulthood, especially in those without other comorbidities [23, 26]. Several factors have been associated with increased disease severity and adverse outcomes. These include obesity, co-infections with HIV or HBV, malignancies, anemia, and high-risk behaviors such as alcohol consumption, intravenous drug use, homelessness, and incarceration [21]. Hepatocellular carcinoma (HCC) in children is estimated to be exceedingly rare, with reports of age-adjusted incidence rates of 0.24 to 0.65/1,000,000 and has been reported almost exclusively in those with cirrhosis [27]. Extrahepatic manifestations are uncommon in children compared to their prevalence in adults. Among them are membranoproliferative glomerulonephritis, thyroid dysfunction, autoimmune thyroid disease, and the presence of non-specific antinuclear antibodies [28]. 

### Screening and Diagnosis

In response to rising HCV prevalence among individuals of reproductive age and persistently low testing rates during pregnancy, the Infectious Diseases Society of America (IDSA) and the American Association for the Study of Liver Diseases (AASLD) advocated for universal HCV screening in pregnant women in 2018 [29]. This recommendation was subsequently endorsed by the U.S. Preventive Services Task Force (USPSTF), the Centers for Disease Control and Prevention (CDC) and American College of Obstetrics and Gynecology [30–32]. Universal screening for HCV during pregnancy can serve to help identify infected mothers as well as facilitate timely treatment and long-term care for them. Additionally, this screening can be used to implement preventative strategies for the infant, including early diagnosis and subsequent monitoring.

Screening recommendations of infants with perinatal exposure to HCV were also updated by the CDC. In 2023 the CDC issued a new statement to screen all exposed infants between 2 to 6 months of age with a nucleic acid test (NAT) for detection of HCV. All infants and children with detectable HCV RNA should be referred to a health care provider with expertise in hepatitis C management. Further follow-up is not required for those with undetectable HCV RNA at age 2 months or older unless clinically warranted. In children aged 7–17 months with perinatal exposure who have previously not been tested, a NAT is also recommended. Additionally, a hepatitis C virus antibody (anti-HCV) test followed by a reflex NAT for HCV RNA is recommended for perinatally exposed children aged ≥ 18 months who previously have not been tested [10].

NAT for HCV RNA cannot be used before age 2 months for diagnosis of perinatal HCV transmission because of false-negative results. HCV RNA during the first weeks of life might indicate contamination with maternal blood or passive transfer of maternal HCV RNA rather than newly established HCV infection of the infant. Anti-HCV tests should not be performed earlier than age 18 months in perinatally exposed children because of passive transfer of maternal antibody [13].

The implementation of these new recommendations for infant HCV testing aligns with the strategy of universal screening during pregnancy. This approach capitalizes on the high rates of medical visits during pregnancy and infancy to identify exposed infants and link them to timely HCV diagnosis and care. This strategy has the potential to reduce the number of infants lost to follow-up and contribute to the ultimate goal of HCV elimination.

### Monitoring of Disease

The initial assessment of children with chronic HCV infection includes exclusion of other causes of liver disease, assessment of disease severity, and detection of extrahepatic manifestations.

Disease staging and progression in pediatric HCV infection can be monitored through regular physical examinations and routine laboratory assessments. Recommended parameters include albumin, serum aminotransferases (AST and ALT), total bilirubin, international normalized ratio (INR), platelet count, and HCV RNA quantification every 6–12 months [21, 29]. INR can be measured at baseline and repeated as needed if patient has not developed cirrhosis.

While liver biopsy remains the gold standard for assessing inflammation and fibrosis, its invasive nature and potential for sampling error has led to a preference for noninvasive alternatives in the era of DAAs. Noninvasive fibrosis markers and ultrasound elastography have demonstrated promise in disease stratification, although further validation is required [33].

Annual abdominal ultrasound and semiannual alpha-fetoprotein (AFP) measurements may be reserved for children with a concern for advanced liver disease, family history of early cirrhosis, HCC, or evidence of rapid disease progression. Standard of care for children with cirrhosis such as endoscopic surveillance for esophageal varices is also indicated.

Testing for concomitant HBV (HBsAg and anti-HBc) and HIV (anti-HIV) are recommended due to shared risk factors. Testing for hepatitis A and B immunity with anti-HAV IgG and anti-HBs respectively, to vaccinate nonimmune children is also recommended [29].

### Treatment

The approval of DAAs in 2011 by the FDA marked a pivotal moment in the management of chronic HCV infection in adults. Subsequently, the high efficacy and tolerability of DAAs have established them as the standard of care for chronic HCV treatment in both adult and pediatric populations, superseding interferon-based regimens. FDA-approved DAA regimens are now available for all children with HCV infection above 3 years of age regardless of HCV genotype or disease severity given that the rate of spontaneous viral clearance is high in the first 3 years of life and the risk of significant liver disease is very low at this age. The AASLD-IDSA guidelines recommend a NAT for HCV RNA to confirm current infection before initiation of DAA therapy [29].

Patients exhibiting extrahepatic manifestations of HCV infection, including cryoglobulinemia, cutaneous eruptions, glomerulonephritis, and thyroid abnormalities, should be considered for early DAA therapy to mitigate the risk of long-term morbidity and mortality. Likewise, individuals with advanced liver fibrosis should be counseled on the benefits of initiating DAA treatment to prevent disease progression [29].

DAAs are mainly categorized into three classes based on their mechanism of action and therapeutic target: Nonstructural proteins 3/4A (NS3/4A) protease inhibitors, NS5B RNA polymerase inhibitors, and NS5A inhibitors.

Currently there are 3 FDA-approved DAA regimens for children with chronic HCV infection.Sofosbuvir/velpatasvir (Epclusa) for a duration of 12 weeks and glecaprevir/pibrentasvir (Mavyret) for a duration of 8 weeks, are oral, pan-genotypic DAA combinations indicated for treatment naive or interferon experienced children and adolescents without cirrhosis or with compensated cirrhosis. DAA-experienced children and adolescents may need a longer treatment duration [29]. Both products contain two DAA drugs that provide a complete regimen for treatment of chronic HCV infection. Epclusa is a combination of sofosbuvir, an HCV nucleotide analog NS5B polymerase inhibitor, and velpatasvir, an HCV NS5A inhibitor. Mavyret is a combination of glecaprevir, an HCV NS3/4A protease inhibitor, and pibrentasvir, an HCV NS5A inhibitor. Weight-based dosing is used for children less than 45 kg or less than 12 years of age. Efficacy for both drugs was based on sustained virologic response (SVR12) defined as undetectable HCV RNA 12 weeks after the end of treatment. The overall SVR12 rate in the clinical trial for Mavyret in children aged 3–12 years was 96% and for adolescents over the age of 12 years was 100% [34, 35]. SVR12 rates for Epclusa were 93% and 91% in adolescents with genotypes 1 and 3, respectively, and 100% in those with genotypes 2, 4, or 6. In children, SVR12 rates were similarly high, with 93% and 91% for genotypes 1 and 3, respectively, and 100% for genotypes 2 and 4 [36].

Ledipasvir/sofosbuvir (Harvoni) is approved for use in children aged 3 to 17 years with genotype 1, 4, 5, or 6 infections for a duration of 12 weeks. As with the above regimens, DAA-experienced children and adolescents may need a longer treatment duration. Harvoni is a combination of Ledipasvir, an NS5A inhibitor, and Sofosbuvir, an HCV nucleotide analog NS5B polymerase inhibitor. The overall SVR12 of three clinical trials in children and adolescents supporting the approval of this regimen was 98% [37–39].

The FDA approved Sofosbuvir plus Ribavirin in 2019 for treatment-naïve or previously treated children aged 3 years and older with genotypes 2 or 3 without cirrhosis or with compensated cirrhosis, however this regimen has been superseded by the availability of pan-genotypic, ribavirin-free DAA regimens.

During treatment, albumin, serum aminotransferases (AST and ALT), total bilirubin, complete blood cell counts, and HCV RNA quantification should be monitored every 4 weeks until completion of treatment. After SVR12 is achieved, children and adolescents with no cirrhosis do not require ongoing monitoring unless clinically warranted. For those with cirrhosis, continued monitoring with regular physical examinations, routine laboratory assessments, and ultrasonography every 6 months is recommended [21].

### Counseling

Demystifying HCV infection in children by providing timely, accurate, and reliable information about viral transmission, natural history, and treatment options is critically important to reassure patients and families. Families and patients often experience shame, fears of potential stigmatization, and extreme caution around disclosing HCV infection due to the association of HCV with IDU and sexual modes of transmission. Caregivers frequently express concerns about disclosing a child's HCV status to school personnel and coaches. Notably, there is no federal or legal mandate for HCV disclosure in the United States, and the CDC lacks specific public guidance on this matter. Given that HCV is not transmitted via casual contact, children with HCV infection should be permitted full participation in all school activities, including sports, without restriction.

Children with chronic hepatitis C are encouraged to maintain a “healthy liver”, which includes maintaining a healthy body weight as obesity has been associated with increased disease severity and adverse outcomes. Similarly, abstinence from alcohol and IDU is strongly advised to minimize disease progression. Providers should also educate patients on proper practices and universal precautions to avoid HCV transmission prior to or during treatment and to avoid reinfection after successfully completing treatment [29].

## Conclusions

The WHO’s newly established global health sector strategy outlines specific actions and targets aimed at eliminating viral hepatitis by 2030 through a reduction in new infections and mortality. Key challenges to achieving this goal include implementing effective screening protocols, bridging the gap between testing and treatment initiation, and ensuring long-term follow-up for affected individuals.

To address the specific issue of pediatric HCV, the United States has implemented strategies to reduce the number of patients lost to follow up and to improve timely care initiation. These strategies include universal screening of pregnant women and NAT for all perinatally exposed infants at 2 to 6 months of age. An important area of further research to help eradicate pediatric HCV is the development of an effective vaccine against HCV to help decrease the rates of mother to child transmission. Developing strategies to increase awareness and screening of adolescents within high-risk populations who have limited access to healthcare is also a priority.

Reducing the cost of treatment at the manufacturing level and improving access to therapy are additional critical components in the global effort to eliminate HCV. Many public and commercial insurance carriers often prioritize treatment for patients with advanced liver disease, a stage of disease infrequently observed in pediatric populations. Consequently, bridging the gap between HCV testing and treatment initiation presents a substantial challenge for healthcare providers. The medication authorization process is often burdensome, characterized by obstacles such as peer-to-peer reviews conducted by insurance physicians lacking HCV expertise and time-consuming requirements that divert practice personnel from patient care. While cost-effectiveness data for pediatric HCV treatment remains limited, existing research supports the initiation of early therapy to enhance quality of life, reduce the life-time risk of complications, and to diminish the overall prevalence of HCV by limiting its transmission through the pediatric and adolescent population [40, 41]. In the absence of a straightforward solution to this challenge, pediatric providers must maintain a comprehensive understanding of current HCV guidelines and approved treatment regimens while actively advocating for expanded access to therapy.

## Key References

- Panagiotakopoulos L, Sandul AL, DHSc, Conners EE, Foster MA, Nelson NP, Wester C, Collaborators*. *CDC recommendations for hepatitis c testing among perinatally exposed infants and children - United States, 2023*. *MMWR Recomm Rep. 2023;72(4):1–21*. *10.15585/mmwr.rr7204a1*.*
  - **Highlights the most current recommendations for testing and monitoring of perinatally exposed children.**
- Bhattacharya D, Aronsohn A, Price J, Lo Re V, AASLD-IDSA HCV Guidance Panel*. *Hepatitis C guidance 2023 update: AASLD-IDSA recommendations for testing, managing, and treating hepatitis C virus infection*. *Clin Infect Dis. 2023. p. ciad319*. *10.1093/cid/ciad319.
  - **Important tool for clinicians for guidance in testing, management and treatment.**
- Balistreri WF, Murray KF, Rosenthal P, Bansal S, Lin CH, Kersey K, Massetto B, Zhu Y, Kanwar B, German P, Svarovskaia E, Brainard DM, Wen J, Gonzalez-Peralta RP, Jonas MM, Schwarz K*. *The safety and effectiveness of ledipasvir-sofosbuvir in adolescents 12-17 years old with hepatitis C virus genotype 1 infection*. *Hepatology. 2017;66(2):371–78*.* 10.1002/hep.28995.
  - **Results of the pivotal trial of ledipasvir-sofosbuvir, demonstrating efficacy and safety of the first FDA approved direct-acting antiviral agent in pediatrics.**

## Data Availability

No datasets were generated or analysed during the current study.
